# Bioelectrical‐Impedance‐Analysis in the Perioperative Nutritional Assessment and Prediction of Complications in Head‐and‐Neck Malignancies

**DOI:** 10.1002/oto2.70046

**Published:** 2025-01-03

**Authors:** Yi Ting Lai, Hui Yee Peh, Hanis Binte Abdul Kadir, Chun Fan Lee, N. Gopalakrishna Iyer, Ting Hway Wong, Gerald Ci An Tay

**Affiliations:** ^1^ NUS Medicine Yong Loo Lin School of Medicine Singapore; ^2^ Singapore General Hospital Singapore; ^3^ Health Services Research Unit Singapore General Hospital Singapore; ^4^ Centre for Quantitative Medicine Duke‐NUS Medical School Singapore; ^5^ Department of Head and Neck Surgery Singapore General Hospital Singapore; ^6^ Department of Head and Neck Surgery National Cancer Centre Singapore; ^7^ Health Services and Systems Research Duke‐NUS Medical School Singapore

**Keywords:** electrical impedance, head and neck neoplasms, malnutrition, nutritional assessment, postoperative complications

## Abstract

**Objective:**

Identification of patients with head‐and‐neck malignancies who are especially vulnerable to malnutrition is critical for optimizing outcomes. The objectives are; to correlate Bioelectrical‐impendence‐analysis (BIA) parameters with Subjective‐Global‐Assessment (SGA) scores, and determine the association of BIA parameters with common perioperative complications in patients undergoing head‐and‐neck surgery.

**Study Design:**

Patients underwent formal SGA scoring and BIA preoperatively in a multidisciplinary allied health clinic.

**Settings:**

This is a cohort study of 61 patients with head‐and‐neck malignancies who were admitted for elective surgery from 2018 to 2019 in a tertiary hospital in Singapore.

**Methods:**

BIA was performed using the Bodystat Quadscan 4000. Kruskal‐Wallis rank sum tests and were performed for associations between SGA and BIA parameters. Wilcoxon rank sum tests and multivariable logistic regression models (Firth's bias reduction method) were performed to evaluate associations between BIA parameters and perioperative complications. Receiver‐operating‐characteristic (ROC) curves were plotted for determination of optimal cut‐off values of phase angle and Wellness marker in detecting malnutrition and perioperative pneumonia using Youden's‐Index (YI).

**Results:**

45 males and 16 females with median age of 62 were included in the study. Significant differences were observed in Wellness Marker (*P* = .006) and phase angle (*P* = .008) among patients in the 3 SGA categories. The Wellness Marker (*P* = .02) was associated with perioperative pneumonia in the univariate analysis. No significant differences were observed for other perioperative complications studied.

**Conclusion:**

BIA shows promise as a preoperative tool, in conjunction with SGA, to detect malnutrition in patients undergoing surgery for head‐and‐neck malignancies and highlight patients at risk of developing perioperative pneumonia.

Despite major advancements in healthcare, malnutrition remains ubiquitous in patients worldwide. Various studies have estimated worrying prevalence rates ranging from 20% to 50%.^1^, ^2^, ^3^ In Singapore, up to one‐third of admitted patients suffer from malnutrition.^4^ The impact of malnutrition cannot be underplayed. Prolonged stays, increased readmission rates, treatment intolerance, and higher in‐patient and long‐term mortality rates are known consequences in such patients globally.^5^, ^6^, ^7^ Malnourished surgical patients have additional intraoperative and postoperative risks including: infections, delayed wound healing, impaired cardiorespiratory functions, and an increased risk of developing severe perioperative complications.^8^ The culmination of these issues generate significant healthcare costs and reduce quality of life.^3^

Patients undergoing surgery for head and neck malignancies are notably vulnerable to developing malnutrition and up to 80% of patients with head and neck cancer are malnourished.^8^ Common symptoms such as loss of appetite, xerostomia, taste alterations, and dysphagia impair deglutition and mastication processes, leading to poor feeding and malnutrition.^9^ Furthermore, inflammation and increased catabolism secondary to malignancy can also severely deplete muscle mass.^10^

Timely and accurate identification of patients at high risk for malnutrition is critical as it allows for early intervention for enhanced outcomes.^3^ Therefore, there is a compelling need for robust, reliable, and quick screening instruments to detect malnutrition. The Subjective Global Assessment (SGA), which relies on the nutritional history and clinical examination for a subjective impression of nutrition status has been a widely endorsed method of nutritional screening^11^, ^12^, ^13^ and is considered by many to be the gold‐standard. However, this method is subjective, time consuming, and requires expertise.^14^

In recent years, bioimpedance analysis (BIA) has been lauded as a portable, safe, reproducible, inexpensive, and noninvasive way of assessing body composition and nutritional status.^15^ Specifically, BIA utilizes predictive equations via measurements of resistance (R) and reactance (Xc) of body tissues along with anthropometric data to generate the phase angle,^16^ a useful parameter reflective of body cell mass and cell membrane functions.^17^ The Wellness marker, on the other hand, is a newly introduced parameter that calculates impedance ratio at various levels of frequency to reflect the generalized state of health of body cells without the need for predictive equations.

The favorable role of BIA in detection of malnutrition and prognosis have been demonstrated in various surgical patient populations such as in cardiac surgery,^18^ gastrointestinal surgery,^19^ and surgical cancer patients^20^ internationally, however, no data exists on the use of bioelectrical impedance for detecting malnutrition in patients undergoing surgery for head and neck malignancies in Singapore. Hence, the current study aims to determine the correlation between SGA scores and BIA parameters, as well as the correlation of BIA parameters with common perioperative complications in patients undergoing head and neck surgery.

## Methods

A prospective study was carried out on 61 patients out of a total of 97 patients scheduled for major head and neck oncological surgery in a tertiary hospital in Singapore from 2018 to 2019. These patients consented to the use of their personal data for research purpose and the study was approved by the institution's ethics committee CIRB No. 2018/2234. Due to patient privacy policies of the institution, the authors are unable to give characteristics of those who refused to be included in the study.

Prior to surgery, patients were evaluated in a preoperative multidisciplinary allied health professional clinic. Subjective Global Assessment (SGA) scores were evaluated by certified dieticians on 61 patients prior to surgery and patients were identified as either well nourished, moderately malnourished, or severely malnourished. The SGA scoring rubric used is provided in the Supplementary data S1. Bioelectrical impedance analysis measuring phase angles and Wellness marker values was performed using the Bodystat Quadscan 4000 in 53 patients in the same month prior to surgery.

All patients were closely monitored and incidences of perioperative complications such as pneumonia, surgical site infections, salivary leak and/or fistula formation and flap complications were recorded until time of discharge.

Statistical analysis was performed with R (version 4.0.2). Kruskal‐Wallis rank sum tests were used to assess associations between BIA parameters and SGA groups, while Wilcoxon rank sum tests evaluated associations between BIA parameters and perioperative complications. For patients undergoing flap surgeries and/or procedures with potential for salivary leaks, specific analyses were conducted for flap complications and salivary leaks, respectively.

Receiver operating characteristic (ROC) curves were plotted to analyse the area under the curves (AUC). Optimal cut‐off values for Wellness marker and phase angle in predicting malnutrition and perioperative pneumonia were determined using the Youden Index (YI). Confidence intervals (CI) for sensitivity, specificity, and AUC were estimated using the Clopper‐Pearson exact method. Multivariable logistic regression models with Firth's bias reduction method^21^ were employed to analyse the four perioperative complications, with BIA parameters, age, smoking status, and tumor site (oral vs non‐oral) as covariates.

## Results

### Patient Characteristics

45 males and 16 females aged ranging from 25 to 88 years old were included in the study. The median age was 62 years [interquartile range [IQR]: 52‐72] and the median BMI was 23.1 kg/m² [IQR: 20.5‐25.8].

Most patients (90.2%) were diagnosed with squamous cell carcinoma in the head and neck region. The remaining patients were diagnosed with adenoid cystic carcinoma, carcinoma ex pleomorphic adenoma, osteosarcoma, and undifferentiated nasopharyngeal carcinoma respectively. The type of operative procedures performed are listed in the Supplementary data S2.

### Prevalence of Malnutrition

Based on the SGA scoring, 24 (40.6%) patients were found to be well‐nourished, 32 (53.1%) patients were moderately malnourished, and 3 (6.3%) patients were severely malnourished.

Patient characteristics and nutritional status determined by SGA are summarized in Table 1.

**Table 1:** Baseline Patient Characteristics

	Statistics
Characteristics	N = 61^a^
Age	62 [52–72]
	N = 61
Gender	
Male	45/61 (73.8%)
Female	16/61 (26.2%)
BMI	23.1 [20.5‐25.8]
	N = 60
Length of stay	14 [10–25]
	N = 61
Diagnosis	
Squamous cell carcinoma	55/61 (90.2%)
Adenoid cystic carcinoma	2/61 (3.3%)
Nasopharyngeal carcinoma	2/61 (3.3%)
Carcinoma ex‐pleomorphic	1/61 (0.02%)
Osteosarcoma	1/61 (0.02%)
Neoplasm sites	
Oral cavity	38/61 (62.3%)
Oropharynx	7/61 (11.5%)
Nasal cavity	3/61 (4.9%)
Nasopharynx	1/61 (1.6%)
Larynx	6/61 (9.9%)
Hypopharynx	2/61 (3.3%)
Salivary gland	2/61 (3.3%)
Thyroid	1/61 (1.6%)
Middle ear	1/61 (1.6%)
T Staging	
T0	1/61 (1.6%)
T1	15/61 (24.6%)
T2	14/61 (23.0%)
T3	13/61 (21.3%)
T4	18/61 (29.5%)
SGA group	
Well nourished	24/59 (40.6%)
Moderately malnourished	32/59 (53.1%)
Severely malnourished	3/59 (6.3%)
BIA parameters	
Phase angle (°)	6.00 [4.60‐7.10]
	N = 53
Wellness marker (Hz)	0.80 [0.78‐0.84]
	N = 52
Fat mass (% of TBW3)	28 [24–36]
	N = 53
Lean mass (% of TBW)	72 [64–76]
	N = 53
Water mass (% of TBW)	59 [56–63]
	N = 53
Body cell mass (kg)	29 [24–32]
	N = 53

Denominators that do not equal the sample sizes are due to missing data.

Abbreviation: SGA, Subjective Global Assessment.

^a^ Median [IQR]; n/N (%).

### BIA Parameters

The median phase angle was 6.00° [IQR: 4.60‐7.10], while the median Wellness marker value was 0.80 Hz [IQR: 0.78‐0.84]. Other parameters recorded from BIA, including individual components of fat, muscle, and water masses as a percentage of total body weight are summarized in Table 1.

The median duration of stay was 14 days [IQR: 10‐25]. In the course of their hospital stay, 21 (34.4%) patients developed perioperative complications. Specifically, 8 developed pneumonia, 12 patients developed surgical site infections, 2 patients developed salivary leak or had fistula formation and 8 patients had flap complications; including 3 patients with arterial thrombosis of the flap vessels, 1 patient with venous thrombosis of the flap vessel, 1 patient with both arterial and venous thrombosis, and 2 patients with flap dehiscence. None of the patients suffered acute myocardial infarctions or cerebrovascular accidents and there were no deaths recorded during admission.

Among the 3 SGA groups, there were statistically significant differences in phase angle (*P* = .008) and the Wellness marker (*P* = .006) measurements, as seen in Table 2.

**Table 2:** Comparison of Phase Angle and Wellness Marker across SGA Groups

Characteristics	Well‐nourished^a^ N = 24	Moderately malnourished^a^ N = 32	Severely malnourished^a^ N = 3	*P* value^b^
Phase angle (°)	6.45 [5.80‐7.73]	5.35 [4.15‐6.33]	4.70 [4.25‐5.55]	.008
	N = 22	N = 28	N = 3	
Wellness marker (Hz)	0.79 [0.76‐0.80]	0.83 [0.79‐0.86]	0.85 [0.82‐0.86]	.006
	N = 22	N = 27	N = 3	

Denominators that do not equal the sample sizes are due to missing data.

^a^ Median [IQR].

^b^ Kruskal‐Wallis rank sum test.

**Figure 1:**
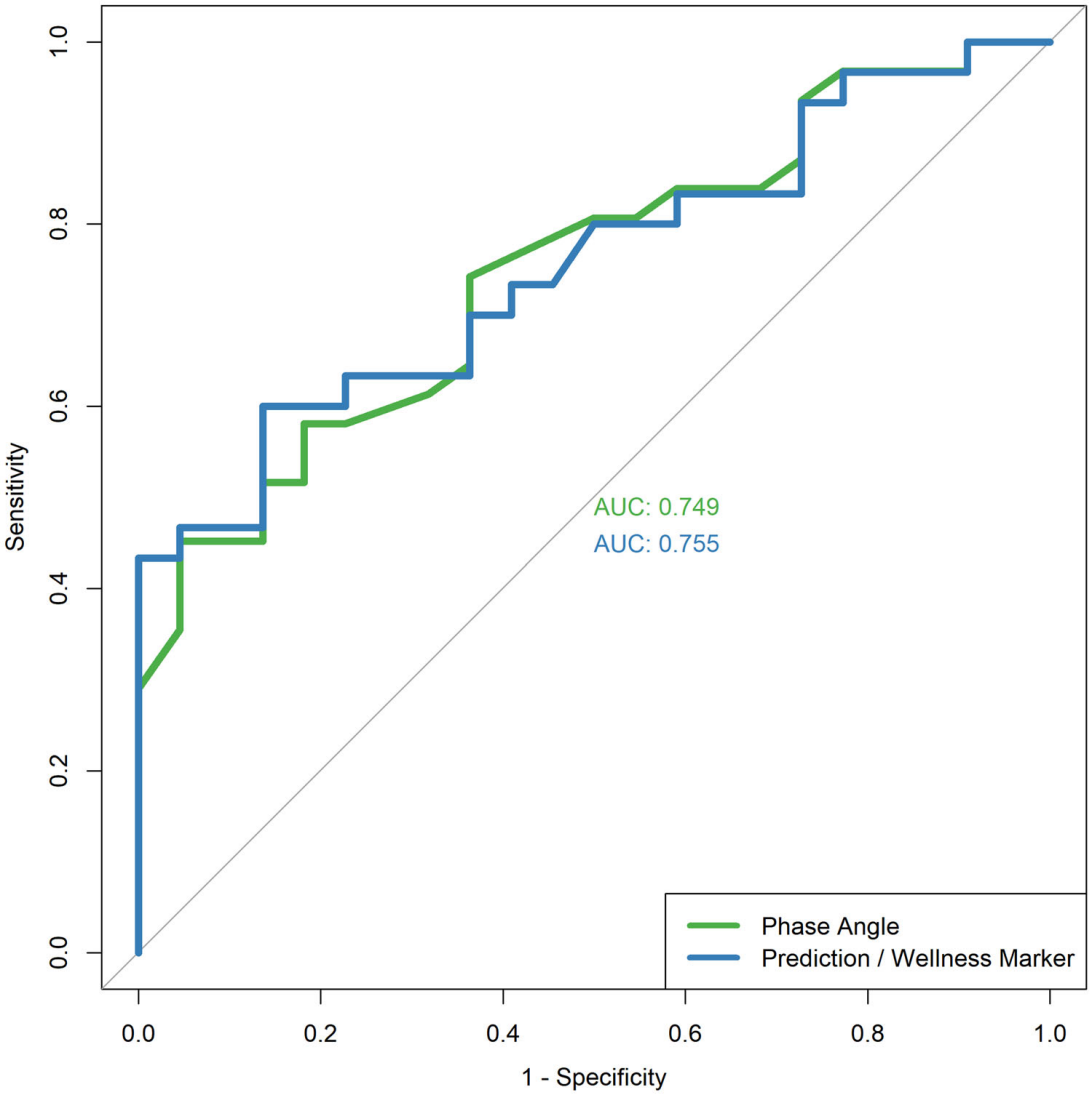
Receiver operating characteristic (ROC) curve of phase angle and Wellness marker for detecting malnutrition.

The optimal phase angle cut‐off in predicting for moderate and severe malnutrition was below 4.7° (sensitivity 45.2% [95% confidence interval [CI] 27.3%‐64.0%], specificity 95.5% [95% CI 77.2%‐99.9%], YI 0.406) while the optimal Wellness marker cut‐off value in predicting for moderate and severe malnutrition was above 0.817. (sensitivity 60.0% [95% CI 40.6%‐77.3%], specificity 86.4% [95% CI 65.1%‐97.1%], YI 0.464). Figure 1 shows the receiver operating characteristic (ROC) curve of phase angle and Wellness marker for detecting malnutrition respectively. Both phase angle (AUC 0.749 [95% CI 0.619‐0.879]) and Wellness marker (AUC 0.755 [95% CI 0.624‐0.885]) provides fair diagnostic accuracy in identifying patients who are malnourished.

Among all perioperative complications recorded, there were statistically significant differences in Wellness marker (*P* = .02) values in patients who developed pneumonia (Table 3). No statistical differences in phase angle and Wellness marker was observed for the development of surgical site infections, salivary leak/fistula, and flap complications respectively.

**Table 3:** Comparison of Phase Angle and Wellness Marker between Patients with and without Perioperative Complications

Perioperative complications	Yes^1^	No^1^	*P* value^2^
Pneumonia			
Phase angle (°)	4.70 [3.55‐5.80]	6.20 [4.90‐7.10]	.062
	N = 8	N = 45	
Wellness marker (Hz)	0.84 [0.83‐0.87]	0.79 [0.77‐0.84]	.020
	N = 7	N = 45	
Surgical site infections			
Phase angle (°)	6.30 [5.65‐6.75]	5.80 [4.30‐7.10]	.5
	N = 11	N = 41	
Wellness marker (Hz)	0.79 [0.78‐0.81]	0.80 [0.77‐0.84]	.5
	N = 11	N = 40	
Salivary leak/fistula			
Phase angle (°)	5.95 [5.73‐6.18]	6.00 [4.63‐7.10]	>.9
	N = 2	N = 50	
Wellness marker (Hz)	0.80 [0.79‐0.81]	0.80 [0.77‐0.84]	>.9
	N = 2	N = 49	
Flap complications			
Phase angle (°)	6.00 [5.25‐6.55]	6.00 [4.60‐7.10]	>.9
	N = 7	N = 45	
Wellness marker (Hz)	0.80 [0.79‐0.84]	0.80 [0.77‐0.84]	.7
	N = 6	N = 45	

Denominators that do not equal the sample sizes are due to missing data.

^a^ Median [IQR].

^b^ Wilcoxon rank sum test.

**Figure 2:**
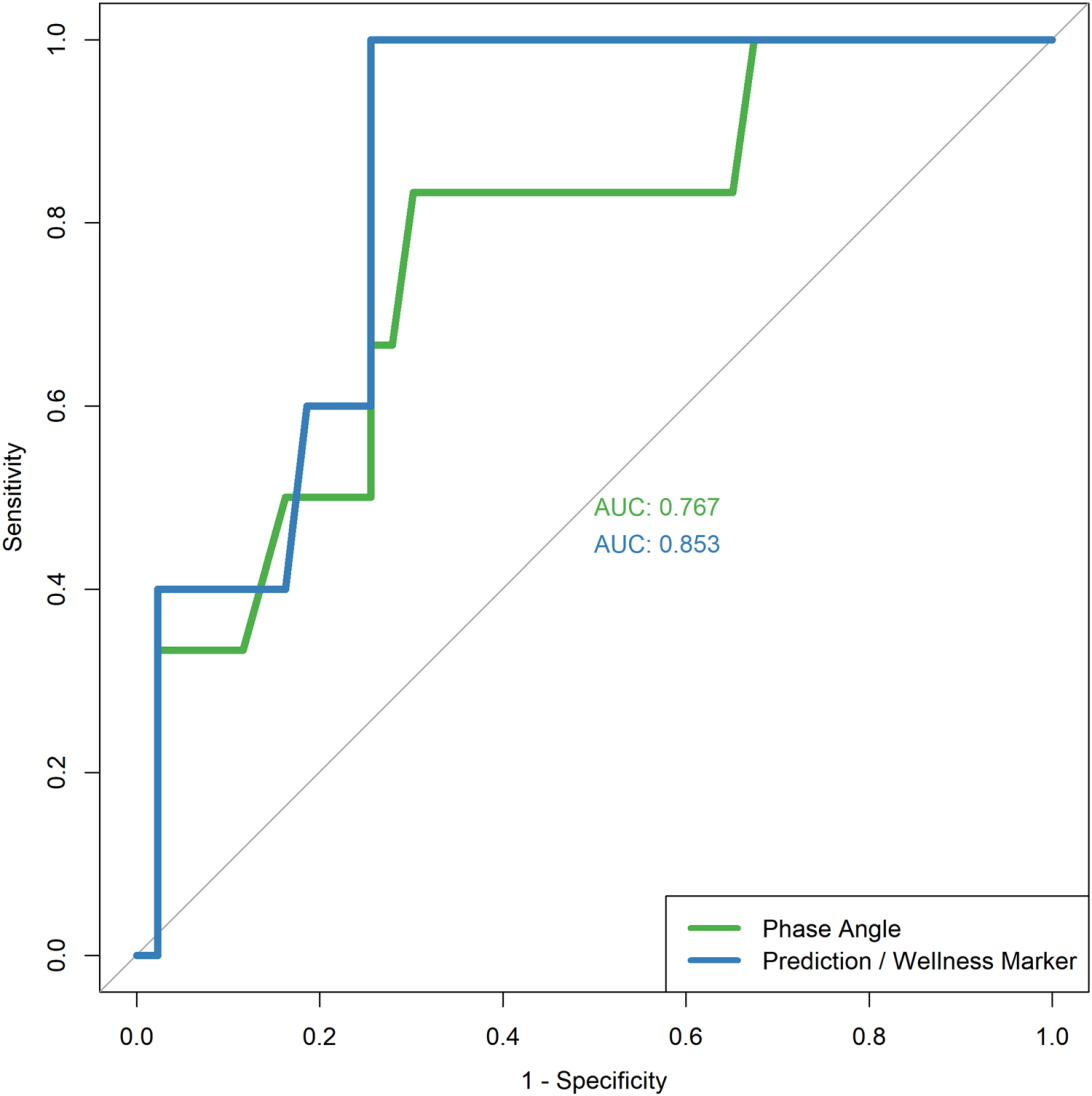
Receiver operating characteristics (ROC) curve for phase angle and Wellness marker in predicting for development of perioperative pneumonia.

The optimal phase angle cut off in predicting for perioperative pneumonia was below 5.5° (sensitivity 83.3% [95% CI 35.9%‐99.6%], specificity 69.8% [95% CI 53.9%‐82.8%], YI 0.531) while the optimal Wellness marker cut off was above 0.829 (sensitivity 100% [95% CI 47.8%‐100%], specificity 74.4% [95% CI 58.8%‐86.5%], YI 0.744). Figure 2 shows the ROC curve for phase angle and Wellness marker in predicting for development of perioperative pneumonia. Both phase angle (AUC 0.767 [95% CI 0.562‐0.973]) and Wellness marker (AUC 0.853 [95% CI 0.724‐0.983]) displayed good discriminating ability in predicting patients with perioperative pneumonia.

In the multivariable analysis, only age showed a statistically significant association with pneumonia risk (OR: 1.16 [95% CI: 1.01‐1.45], *P* = .025, per year increase) (Table 4). Neither phase angle nor Wellness marker demonstrated statistically significant associations with pneumonia risk, although Wellness marker suggested a potential increase in risk (OR: 3.67 [95% CI: 0.70‐32.3], *P* = .15). No statistically significant associations were found for any variables with the other perioperative complications examined.

**Table 4:** Multivariable Models for (a) Pneumonia, (b) Surgical Site Infections, (c) Salivary Leak/Fistula, and (d) Flap Complications using Logistic Regression Model with Firth's Bias Reduction Method, with BIA Parameters as the Independent Variables

	(a) Pneumonia		(b) Surgical site infections		(c) Salivary leak/fistula		(d) Flap complications	
Characteristics	OR (95% CI)	*P* value	OR (95% CI)	*P* value	OR (95% CI)	*P* value	OR (95% CI)	*P* value
Phase angle, ° (per 0.01 unit increase)	1.04 (0.99, 1.10)	.2	1.01 (0.98, 1.04)	.6	0.99 (0.88, 1.06)	.8	1.01 (0.96, 1.05)	.7
Wellness marker, Hz (per 0.01 unit increase)	3.67 (0.70, 32.3)	.15	1.32 (0.57, 3.66)	.5	0.83 (0.03, 9.39)	.9	1.57 (0.37, 6.26)	.5
Age (per 1 year increase)	1.16 (1.01, 1.45)	.025	0.99 (0.93, 1.05)	.7	0.93 (0.69, 1.05)	.3	0.95 (0.87, 1.02)	.14
Smoking status (yes vs no)	0.86 (0.12, 6.43)	.9	2.73 (0.64, 15.9)	.2	4.50 (0.30, 1.002)	.3	0.76 (0.14, 4.47)	.8
Tumor site (oral vs non‐oral)	0.53 (0.06, 4.12)	.5	0.74 (0.18, 3.09)	.7	1.49 (0.08, 151)	.8	1.12 (0.16, 8.83)	>.9

Abbreviations: CI, confidence interval; OR, odds ratio.

## Discussion

In our study, we found that a sizeable proportion of patients undergoing surgery for head and neck malignancies were malnourished (57.4%). Other studies have similarly reported high incidences of malnutrition in head and neck cancer patients, with prevalence rates ranging from 25% to 80%.^8^, ^22^, ^23^

Patients suffering from head and neck malignancies are especially susceptible to developing malnutrition because of impaired metabolism from disease processes, poor oral intake due to symptoms such as dysphagia, anorexia, mucositis, xerostomia, and taste alterations.^24^, ^25^ Furthermore, many patients suffer from chronic malnutrition associated with alcohol and tobacco use, compounding the present issue.^8^

Multiple cohort studies have demonstrated association between SGA scores and BIA parameters in healthy subjects,^26^ hospital in‐patients,^15^ and surgical patients.^18^, ^20^, ^27^ Notably, Małecka‐Massalska et al demonstrated significantly lower phase angles among malnourished patients in a cohort of 75 newly diagnosed head and neck cancer patients.^28^ Our results support these findings.

Bioimpedance analysis can provide valuable information on nutritional status via the determination of phase angles, which is a measure of cell membrane integrity and vitality and hence reliably reflects cellular health^29^ and allows for accurate nutritional assessment of patients with altered hydration status.^30^, ^31^ However, reference values of phase angles vary according to sex, age, BMI, and disease processes.^32^ Therefore, interpretation of phase angles should be population‐specific, as body composition varies between populations. For instance, higher body fat percentages in Asians compared to Caucasian counterparts of the same BMI presents significant challenges in comparing between studies. In the present study, we established optimal cut‐offs specific to head and neck surgical patients in an Asian context. It may also be relevant for use in patients with head and neck squamous cell carcinoma, as these patients form the majority (90.2%) in our study population. Further studies will be required to validate and refine the values reported. The Wellness marker, also known as impedance ratio, is a relatively newer parameter introduced only in more modern BIA systems and differs from phase angle measurements in that no predictive equations are required. Thus, Wellness marker values can be compared across populations as it is not affected by the subject's weight, gender, age nor BMI values.^33^ Preliminary studies have also reported the wellness marker as a more robust marker of nutrition and disease severity.^34^ However, only limited studies in literature exists for the clinical utility of the wellness marker currently.^35^

Our study also established that BIA predicts development of perioperative pneumonia. Several other studies have also demonstrated the role of BIA in predicting complications.^19^ In our study population, BIA was predictive only for perioperative pneumonia amongst other complications studied, namely salivary leak, wound infections, and flap complications. BIA does not readily predict for the latter complications as they are often multifactorial and are likely more dependent on risk factors such as type of surgery, previous irradiation, and other technical factors, which varies greatly between individuals. On the contrary, malnutrition has been strongly associated with pneumonia; protein‐calorie malnutrition has been found to impair pulmonary cell‐mediated immunity processes and clearance of pathogens, resulting in increased incidence, severity, and duration of pulmonary infections in malnourished individuals.^36^ Besides perioperative complications, studies focused on head and neck cancer patients have also showed correlation of BIA parameters with prolonged hospital stay,^37^ survival rates,^9^, ^10^, ^38^ and radiotherapy outcomes.^39^

The value of BIA has evolved greatly since it was validated for use in assessing human body composition in 1983.^40^ Since then, it has been well regarded as an objective, convenient, non‐invasive, safe, portable, and inexpensive tool for assessment of malnutrition and more.^35^, ^41^ BIA may also be particularly useful in determining nutritional status and prognosis in cancer patients, as malignancy alter homeostatic processes and alters body composition.^42^ Notably, the United Kingdom National Multidisciplinary Guidelines for Head and Neck Cancer, Sixth Edition, specifies BIA as an objective and desirable form of body composition measurement in the general pre‐treatment recommendations for head and neck cancer patients.^43^ However, BIA measures cannot be extrapolated to other populations and requires individuals to be relatively well‐hydrated for accurate measurements.^42^ Due to limited validation studies in hospitalized patients and variability between BIA devices and body compartments estimated within studies, the prevailing American Society for Parenteral and Enteral Nutrition (ASPEN) clinical guidelines have not yet recommended BIA for use in clinical populations.^44^ The SGA, on the other hand, is a validated method that is commonly employed in clinical settings. While it has achieved wide acceptance in its use globally,^45^ SGA lacks sensitivity to detect acute changes in nutritional status.^46^, ^47^ Furthermore, it remains a subjective tool with inter‐observer variability which greatly impairs its applicability on a continuum.^12^, ^48^ Unlike BIA, SGA is also time‐consuming and requires trained professionals for reliable administration.^14^ Therefore, we propose the use of BIA as a useful and convenient adjunct with other measures of nutrition in identifying and predicting for malnutrition and perioperative pneumonia.

The limitations of our study include a small patient cohort size from a single institution and the lack of long‐term follow‐up data. Moreover, the small patient cohort size may be a limiting factor in the multivariable analysis, especially when considering multiple variables simultaneously. In this context, the study may be underpowered to detect significant associations, potentially affecting the robustness of our findings. Due to miscommunications relating to availability of patient consent and study recruitment, few patients did not undergo preoperative SGA and BIA scoring. The presence of existing comorbidities, neoadjuvant radiation therapy, and varying operative durations may also confound the current analysis. In addition, the type of perioperative pneumonia and subsequent interventions these patients received have not been discussed within the scope of this paper. Further research may be required to determine cost‐effectiveness and practicality of BIA for routine use in an institutional setting.

## Conclusion

Bioelectrical Impedance Analysis is associated with Subjective Global Assessment, and can be used in assessing preoperative nutritional status for patients undergoing surgery for head and neck malignancies. BIA shows promise as a preoperative tool, in conjunction with SGA to detect malnutrition in patients undergoing head and neck surgery and highlight patients at risk of developing perioperative pneumonia.

## Author Contributions

**Ting Hway Wong**, design, manuscript; **Hui Yee Pei**, design, conduct, manuscript; **Gerald Ci An Tay**, design, conduct, manuscript; **N. Gopalakrishna Iyer**, design; **Hanis Binte Abdul Kadir**, analysis; **Chun Fan Lee**, analysis; **Yi Ting Lai**, manuscript.

## Disclosures

### Competing interests

None.

### Funding source

This study was made possible by funding from the ACP Seed Grant. The ACP seed grant is funded by the SingHealth Duke‐NUS Academic Medical Centre, facilitated by the Joint Office of Academic Medicine. It is an initiative of Surgery Academic Clinical Program, sited at the Singapore General Hospital. The authors hereby declare there is no conflict of interest.

## Supporting information

Supporting information.

Supporting information.
