# Supplementary material for: Bioelectrical‐Impedance‐Analysis in the Perioperative Nutritional Assessment and Prediction of Complications in Head‐and‐Neck Malignancies
Source: OTO Open. 2025 Jan 3;9(1):e70046. doi: 10.1002/oto2.70046 (PMC11696890; doi:10.1002/oto2.70046)
Supplement: Supplementary file 1 — Supporting information. [file OTO2-9-e70046-s001.pdf]

Subjective Global Assessment Form

Patient's sticker

Part 1: History

	A	B	C
Weight Change<br>Weight change in past 6 months: _______kg	□ <5% loss	□ 5 – 10% loss	□ > 10% loss
Change in past 2 weeks	□ increase	□ no change	□ decrease
Dietary Intake Change<br>Overall change: ___ no change ___ change Duration: ____ weeks	□	□	□
Type of change: ___ suboptimal solid diet ___ full liquid diet<br>___ hypocaloric liquid ___ starvation	□	□	□
GI symptoms change (persisted >2 weeks)<br>___ none ___ nausea ___ vomiting ___diarrhoea ___ anorexia	□	□	□
Functional capacity<br>Overall improvement:	□ none	□ moderate	□ severe
Change in past 2 weeks:	□ improved	□ no change	□ regressed
Disease and its relation to nutritional requirements<br>Metabolic demands, stress level: ___ no ___ low ___ moderate ___ high	□	□	□

Part 2: Physical Examination

	A	B	C
Loss of subcutaneous fat (triceps, chest)	□	□	□
Muscle wasting (quadriceps, deltoids)	□	□	□
Edema (ankle, sacral)	□	□	□
Ascites	□	□	□

Part 3: Overall SGA rating (select one)

□ **A**	Well nourished
□ **B**	Moderately (or suspected of being) malnourished
□ **C**	Severely malnourished
