# Supplementary material for: Bioelectrical‐Impedance‐Analysis in the Perioperative Nutritional Assessment and Prediction of Complications in Head‐and‐Neck Malignancies
Source: OTO Open. 2025 Jan 3;9(1):e70046. doi: 10.1002/oto2.70046 (PMC11696890; doi:10.1002/oto2.70046)
Supplement: Supplementary file 2 — Supporting information. [file OTO2-9-e70046-s002.docx]

| **SN** | **NATURE OF OP** |
| --- | --- |
| N001 | TRACHEOSTOMY, LEFT NECK DISSECTION, RIGHT HEMIGLOSSECTOMY, FREE FLAP |
| N002 | TOTAL GLOSSOLARYNGECTOMY, BIL NECK DISSECTION |
| N003 | - |
| N004 | WEDGE RESECTION OF R TONGUE TUMOUR |
| N005 | PARTIAL PHARYGOLARYNGECTOMY, BIL NECK DISSECTION, FREE FLAP |
| N006 | TRANSORAL ROBOTIC SURGICAL RESECTION OF RIGHT SOFT PALATE TUMOUR, RIGHT NECK DISSECTION |
| N007 | LEFT PARTIAL GLOSSECTOMY, LEFT NECK DISSECTION |
| N008 | FOM RESECTION, BIL NECK DISSECTION, TRACHEOSTOMY, FREE FLAP |
| N009 | HEMIMAXILLECTOMY, OBTURATOR INSERTION |
| N010 | TPL WITH BILATERAL NECK DISSECTION, END TRACHEOSTOMY, TEP AND INSERTION |
| N011 | LEFT PAROTIDECTOMY, LEFT NECK DISSECTION, LEFT MANDIBULECTOMY, FREE FLAP |
| N012 | RIGHT HEMIGLOSSECTOMY, RIGHT NECK DISSECTION |
| N013 | TOTAL LARYNGECTOMY, BIL NECK DISSECTION |
| N014 | WIDE RESECTION, MARGINAL MANDIBULECTOMY, RIGHT NECK DISSECTION, FREE FLAP |
| N015 | RIGHT HEMIGLOSSECTOMY, RIGHT NECK DISSECTION |
| N016 | SUBTOTAL GLOSSECTOMY, BIL NECK DISSECTION, TRACHEOSTOMY, FREE FLAP |
| N017 | TOTAL LARYNGECTOMY, PRIMARY CLOSURE, BIL NECK DISSECTION, PRIMARY INSERTION OF TEP |
| N018 | RIGHT PARTIAL MAXILLECTOMY, RIGHT NECK DISSECTION, OBTURATOR |
| N019 | LEFT HEMI, LEFT NECK DISSECTION |
| N020 | TOTAL THYROIDECTOMY, LARYNGOPHARYNGECTOMY, BIL NECK DISSECTION, FREE FLAP RECON |
| N021 | LEFT PARTIAL GLOSSECTOMY, LEFT NECK DISSECTION |
| N022 | TOTAL LARYNGECTOMY, BIL NECK DISSECTION |
| N023 | TOTAL LARYNGECTOMY, PHARYNGEAL RECON, FLAP |
| N024 | LEFT SUBTOTAL TEMPORAL BONE RESECTION, LEFT PAROTIDECTOMY, LEFT NECK DISSECTION, FLAP |
| N025 | TRACHY, LEFT NECK DISSECTION, LEFT SEGMENTAL MANDIBULECTOMY, FREE FLAP RECON |
| N026 | RIGHT NECK DISSECTION, RIGHT LATERAL OROPHARYNGECTOMY |
| N027 | PEG INSERTION, TRACHY, BIL NECK DISSECTION, BIL INFERIOR MAXILLECTOMY, OBTURATOR |
| N028 | PARTIAL GLOSSECTOMY, NECK DISSECTION, TRACHY, FREE FLAP |
| N029 | PARTIAL GLOSSECTOMY, BIL NECK DISSECTION, TRACHY |
| N030 | TRACHY, LEFT NECK EXPLORATION, MANDIBULECTOMY, LATERAL OROPHARYNGECTOMY, FREE FLAP |
| N031 | RIGHT SEGMENTAL MANDIBULECTOMY, FREE FLAP, OSTEOTOMY, RECONTOURING |
| N032 | TRACHY, RIGHT NECK LN EXCISIONAL BIOPSY, LEFT NECK DISSECTION, LEFT SEGMENTAL MANDIBULECTOMY, LEFT POSTERIOR MAXILLARY ALVEOLECTOMY, WIDE EXCISION OF LEFT OROPHARYNGEAL TUMOUR, FREE FLAP |
| N033 | WIDE EXCISION OF RIGHT BUCCAL SCC, RIGHT NECK DISSECTION, TRACHY, FREE FLAP |
| N034 | RIGHT PARTIAL GLOSSECTOMY AND RIGHT NECK DISSECTION |
| N035 | TRACHY, LIGATION OF LINGUAL ARTERY, LEFT PARTIAL GLOSSECTOMY, LATERAL OROPHARYNGECTOMY, BUCCAL FAT PAD CLOSURE |
| N036 | SUBTOTAL MAXILLECTOMY AND RIGHT NECK DISSECTION |
| N037 | OPEN TRACHY AND OPEN LEFT VOCAL TYPE III CORDECTOMY |
| N038 | TRACHY, BIL NECK DISSECTION, LEFT MAXILLARY SWING, NASOPHARYNGECTOMY, FREE FLAP |
| N039 | TRACHY, BIL NECK DISSECTION, RIGHT HEMI |
| N040 | OPEN CRANIOFACIAL RESECTION, TRACHY, RIGHT ORBITAL EXENERATION, RIGHT NECK DISSECTION, RIGHT MAXILLECTOMY WITH RESECTION OF FRONTAL BONE, FREE FLAP OF ORBITAL DEFECT AND PERICRANIAL FLAP |
| N041 | TRACHY, WIDE RESECTION OF FOM TUMOUR, SEGMENTAL MANDIBULECTOMY, BIL NECK DISSECTION, FREE FLAP |
| N042 | TRACHY, COMPOSITE RESECTION OF RIGHT HEMIMANDIBULE, INFERIOR PARTIAL MAXILLECTOMY, RIGHT NECK DISSECTION, FREE FLAP |
| N043 | LEFT SUBTOTAL GLOSSECTOMY, FLOOR OF MOUTH RESECTION, PARTIAL THYOIDECTOMY, BIL NECK DISSECTION, FREE FLAP |
| N044 | RIGHT SUBTOTAL MAXILLECTOMY, RIGHT SELECTIVE NECK DISSECTION, OBTURATOR |
| N045 | LEFT PARTIAL GLOSSECTOMY, LEFT NECK DISSECTION |
| N046 | TOTAL PHARYNGOLARYNGECTOMY, BIL NECK DISSECTION, PRIMARY TEP, PEG |
| N047 | TRACHY, BIL SUPRAOMOHYOID NECK DISSECTION, BIL INFERIOR MAXILLECTOMY, DEBULKING OF LEFT NPC MASS. OBTURATOR |
| N048 | TRACHY, TORS HYPOPHARYNGECTOMY, LEFT NECK DISSECTION, FREE FLAP |
| N049 | TRACHY, RIGHT NECK DISSECTION, RIGHT HEMIGLOSSECTOMY, FREE FLAP |
| N050 | WIDE EXCISION, LEFT UPPER ALVEOLECTOMY TRACHY, LEFT NECK DISSECTION, FREE FLAP |
| N051 | TRACHY, LEFT NECK DISSECTION, LEFT HEMIGLOSSECTOMY, FREE FLAP, REMOVAL OF TEETH |
| N052 | TRACHY, RIGHT NECK DISSECTION, RIGHT SEGMENTAL MANDIBULECTOMY, FREE FLAP |
| N053 | RIGHT TOTAL PAROTIDECTOMY, LEFT SUPERFICIAL PAROTIDECTOMY, BIL SUPRAOMOHYOID NECK DISSECTION |
| N054 | LEFT GLOSSECTOMY, LEFT NECK DISSECTION |
| N055 | MARGINAL MANDIBULECTOMY, BIL NECK DISSECTION, SPLIT SKIN GRAFT |
| N056 | Tracheostomy, segmental mandibulectomy, bilateral supraomohyoid neck dissection and Free fibula Flap reconstruction |
| N057 | LEFT HEMIGLOSSECTOMY, SUPRAOMOHYOID NECK DISSECTION, |
| N058 | Tracheostomy, Segmental mandibulectomy, bilateral supraomohyoid neck dissection and Free fibula Flap reconstruction |
| N059 | Tracheostomy, Right partial glossectomy, right neck dissection level I-V and radial forearm free flap reconstruction |
| N060 | Bilateral inferior maxillectomy and reconstruction with radial forearm free flap and split skin graft from leg to radial forearm |
| N061 | Right neck dissection level I-IV, wide excision right buccal tumour, marginal mandibulectomy and buccal fat pad closure |
